# Effect of organic acids on fermentation quality and microbiota of horseshoe residue and corn protein powder

**DOI:** 10.1186/s13568-024-01686-4

**Published:** 2024-05-18

**Authors:** Chao Zhao, Yue Li, Qiong Chen, Yongqing Guo, Baoli Sun, Dewu Liu

**Affiliations:** College of Animal Science, South China Agricultural University, Guangzhou, 510642 PR China

**Keywords:** Chinese water chestnut, Corn gluten meal, Malic acid, Citric acid, Mixed silage, Microbial community

## Abstract

This experiment aimed to investigate the impact of malic acid (MA) and citric acid (CA) on the nutritional composition, fermentation quality, rumen degradation rate, and microbial diversity of a mixture of apple pomace and corn protein powder during ensiling. The experiment used apple pomace and corn protein powder as raw materials, with four groups: control group (CON), malic acid treatment group (MA, 10 g/kg), citric acid treatment group (CA, 10 g/kg), and citric acid + malic acid treatment group (MA, 10 g/kg + CA, 10 g/kg). Each group has 3 replicates, with 2 repetitions in parallel, subjected to mixed ensiling for 60 days. The results indicated: (1) Compared to the CON group, the crude protein content significantly increased in the MA, CA, and MA + CA groups (*p* < 0.05), with the highest content observed in the MA + CA group. The addition of MA and CA effectively reduced the water-soluble carbohydrate (WSC) content (*p* < 0.05). Simultaneously, the CA group showed a decreasing trend in NDFom and hemicellulose content (*p* = 0.08; *p* = 0.09). (2) Compared to the CON group, the pH significantly decreased in the MA, CA, and MA + CA groups (*p* < 0.01), and the three treatment groups exhibited a significant increase in lactic acid and acetic acid content (*p* < 0.01). The quantity of lactic acid bacteria increased significantly (*p* < 0.01), with the MA + CA group showing a more significant increase than the MA and CA groups (*p* < 0.05). (3) Compared to the CON group, the in situ dry matter disappearance (ISDMD) significantly increased in the MA, CA, and MA + CA groups (*p* < 0.05). All three treatment groups showed highly significant differences in in situ crude protein disappearance (ISCPD) compared to the CON group (*p* < 0.01). (4) Good’s Coverage for all experimental groups was greater than 0.99, meeting the conditions for subsequent sequencing. Compared to the CON group, the Shannon index significantly increased in the CA group (*p* < 0.01), and the Simpson index increased significantly in the MA group (*p* < 0.05). However, there was no significant difference in the Chao index among the three treatment groups and the CON group (*p* > 0.05). At the genus level, the abundance of Lentilactobacillus in the MA, CA, and MA + CA groups was significantly higher than in the control group (*p* < 0.05). *PICRUSt* prediction results indicated that the metabolic functional microbial groups in the CA and MA treatment groups were significantly higher than in the CON group (*p* < 0.05), suggesting that the addition of MA or CA could reduce the loss of nutritional components such as protein and carbohydrates in mixed ensilage. In conclusion, the addition of malic acid and citric acid to a mixture of apple pomace and corn protein powder during ensiling reduces nutritional losses, improves fermentation quality and rumen degradation rate, enhances the diversity of the microbial community in ensiled feed, and improves microbial structure. The combined addition of malic acid and citric acid demonstrates a superior effect.

## Introduction

Chinese water chestnut (Eleocharis tuberosa), also known as “horse’s hoof”, is a vegetable with high nutritional and health care value, with white and sweet flesh, and is clinically used to treat coughs, sore throats, and urinary difficulties (Li et al. 2022). In folk medicine, water chestnut is also used to treat chronic nephritis, pharyngitis and enteritis (You et al. 2007). Water chestnuts are regarded as a nutritious delicacy in catering (Hodge 1956). However, water chestnut by-products are produced while consuming water chestnut. About 20% of the total mass of water chestnut (w/w) is made up of Chinese water chestnut residue (CWCR),a by-product of water chestnut consumption that is often discarded as waste (Xu et al. 2018). This causes great pollution to the environment. The results of previous studies showed that CWCR could be used as a biosorbent to remove the material from wastewater (Khan et al. 2013). Meanwhile, CWCR contains a large number of healthful bioactive components, such as phenolic compounds, flavonoids, sterols, and polysaccharides, whose small-molecule structures and bioactivities have been widely reported (El-Naggar et al. 2018). This indicates that CWCR has the potential to be used as a natural functional feed. Therefore, CWCR development and utilization has become one of the hotspots for scientists’ research. But even with silage, the high moisture content of CWCR makes it difficult to preserve for a long period of time. Moreover, the high moisture content of silage creates a lot of undesirable microorganisms that affect feed fermentation quality. Previous researchers reported that mixing high-moisture by-products with dry crops is an effective way to solve the problem of high-moisture silage (Zhao et al. 2021).

A significant by-product of wet processing corn starch is corn gluten meal (CGM), with a protein content of about 60%, rich in a variety of amino acids such as leucine, alanine and serine (Loy and Lundy 2019). However, it has several issues, including acid imbalance (Gümü et al. 2020), low essential amino acid content (VILA et al. 2020), and low digestibility (Jun et al. 2020). This high protein feed widely used in livestock production is difficult to be absorbed and utilized by the gut after intake, resulting in unsatisfactory utilization (Brown et al. 2022). Therefore, proper pretreatment is necessary to improve the effective utilization of CGM. A common way to improve the utilization of CGM is enzymatic treatment, which effectively reduces the production of antinutritional factors in CGM and thus improves the hydrolysis and digestibility of the substrate. However, a bitter taste and poor heat tolerance due to enzymatic digestion that impacts the palatability of CGM, which in turn causes intestinal immune stress and livestock performance. Therefore, enzymatic digestion faces challenges such as variable strain performance and low tolerance. Currently, adding organic acids directly to the silage seems like a potential way to accelerate substrate fermentation in conjunction with feedstock microbes and reduce the fermentation cycle (da Silva et al. 2012). As a result, this process breaks down the substrate into smaller peptides that are more easily absorbed.

Nowadays, the production of silage makes extensive use of additives. These can be divided into four categories based on their purpose and impact: fermentation inhibitors, fermentation enhancers, aerobic bacterial inhibitors and nutritional additives. Organic acids are currently widely used as silage fermentation inhibitors in silage production because they may quickly lower the pH value at the first stage of mixed silage and inhibit enzymes and microbial activity (Guo et al. 2007). Malic acid (MA) and Citric acid (CA) are important organic acids produced in the process of sugar metabolism of living organisms, which are widely existed in the fruits and vegetables in nature. They are also important intermediate products of the tricarboxylic acid cycle of living organism metabolism and a byproduct of the CO_2_ fixation reaction (Ke et al. 2017). In production, MA and CA are used as a good new silage additives with strong antioxidant effect, which can rapidly reduce pH, decrease protein hydrolysis, accelerate the growth of lactic acid bacteria, and inhibit the growth of yeasts and molds. In addition, lactic acid bacteria can grow and multiply more quickly when CA and MA are used as a carbohydrate source to supply energy for microbial activity. The results of Ke et al. showed that CA and MA with lactic acid bacteria could improve the fermentation quality of alfalfa silage (Ke et al. 2018). However, little is known about how CA and MA affect the microbial community and fermentation quality of mixed CWCR and CGM silage.

In order to further improve the feed utilization of CWCR and CGM, the study was conducted to combine MA and CA with CWCR and CGM as raw materials. A two-factor test was used to determine the synergistic effect of the two. Additionally, the chemical composition, fermentation quality, rumen degradation, and microbial compartments were analyzed to obtain the synergistic fermentation products of organic acids with higher digestibility. Finally, the study provided technological support for the fermentation of CWCR and CGM by organic acids and laid the theoretical foundation for their production, processing, development, and use.

## Materials and methods

### Silage preparation

CWCR and CGM were purchased from Guangdong Province Wenshi Food Group Co. Ltd (112°14’E, 22°44’N), and MA and CA were provided by the Herbivore Laboratory of South China Agricultural University. CWCR and CGM were mixed well in a small blender at a ratio of 76:24 to adjust the moisture to 70%. Adopting a two factor experimental design. There were four experimental groups: control (CON), Malic acid treatment (MA), Citric acid treatment (CA) and mixed CA + MA treatment. CA and MA addition amounts are both 10 g/kg. Both additives were dissolved in 20 mL of distilled water and sprayed uniformly in 1 kg of mixed silage using a small spray bottle, and the CON group was sprayed with an equal amount of distilled water. Placement of well-mixed mixed silage in food-grade fermentation bags (350 × 450 mm) with one-way venting valves, sealed using a XH-1200BL vacuum packaging machine (Xuheng Technology, China) to ensure anaerobic fermentation, and placed in a silage room (25 °C ± 3℃) with a thermostatic system to ferment for 60 days. Each test group was prepared with 3 replicates per group, 2 parallels per replicate, and the weight of the fermentation bag was recorded for each replicate. After completion of fermentation 3 samples were removed for testing. One sample was placed at -80 °C for microbiota and microbial enumeration, the second sample was used for chemical composition and fermentation quality measurements, and the third sample was used for in vitro simulated digestion and rumen degradation measurements.

### Fermentation quality analysis

Take out the mixed silage sample after 60 days of fermentation, and place it for 15 min at 105℃. After that, immediately dry it for 48 h at 65℃. The dried samples were pulverized and passed through a 40-mesh sieve by means of a SWLF-600 model grinder (Xulang Technology, China). The dry matter (DM) content of each group was determined and the dry matter recovery (DMR) was determined according to the method described by AOAC. Crude protein (CP) content was determined using a fully automated Kjeldahl analyzer model 6800 (Foss, Sweden) (Hasan 2015). Neutral detergent fiber (NDF), acid detergent fiber (ADF) and acid detergent lignin (ADL) of the samples were determined using a fiber analyzer model A200i (ANKOM, USA) (Van Soest et al. 1991), and subsequently the residues of NDF and ADF were placed in a muffle furnace model EL-F (CARBOLITE GERO, Germany) at 550 °C for 2 h to obtain NDFom and ADFom. The cellulose content was calculated using the difference between ADFom and ADL, and the hemifiber content was calculated from the difference between NDFom and ADFom. The content of WSC was determined by colorimetric method using 3,5-dinitrosalicylic acid (Thomas 1977). Hao et al’s method was used to determine starch content (Jiang and Lu 2018).

### Chemical composition analysis

A sample of 15 g of mixed silage was taken, added with 135 mL of distilled water, and incubated in an air shaker at 4 °C overnight, and then filtered using four layers of gauze and qualitative filter paper, and the filtrate was immediately measured using a pH meter (Sartorius PB-10), and then centrifuged at 12,000 r/min for 15 min at 4 °C, and then filtered through a 0.22 μm membrane. The resultant filtrate was divided into two portions, one for the determination of acetic acid (AA), propionic acid (PA) and butyric acid (BA) in the filtrate using an Intuvo 9000 gas chromatography system (Agilent, USA), and the other for the determination of NH_3_-N using the colorimetric method of phenol-sodium hypochlorite (Broderick and Kang 1980).

### Microbiological analysis

The samples were removed from the refrigerator at -80 °C and the mixed silage was analyzed microbiologically by plate counting (Olsen and Bakken 1987). To get the 10^− 1^ dilution, first weigh 10 g of the sample, add 90 mL of sterilized distilled water, mix the sample thoroughly in a sterile polyethylene bag, then tap with a sterile homogenizer for 2 min, the 10^− 1^-10^− 9^ gradient was made by successive dilution with sterilized distilled water at a ratio of 1:9, and the 3 optimal gradients, namely, 10^− 2^, 10^− 4^, and 10^− 6^, were chosen to spread the dilution on the medium, and the number of microorganisms was determined according to the corresponding dilution times. Plate counting method was used to count the microorganisms, and the number of microorganisms was determined by conversion according to the corresponding dilution times (Chen et al. 2012). The results were summarized as follows: the number of *lactobacilli* was determined using BLM medium (De Man Rogosa, Thermo Fisher Scientific). For coliform counts, BLB medium (Blue light broth agar, Thermo Fisher Scientific) was used and incubated for 48 h at 30 °C. For yeast and mold counts, PDA medium (Potato dextrose agar, Thermo Fisher Scientific) was used and incubated at 30 °C for 24 h (Cai et al. 2011).

### Measurement of rumen degradation characteristics

The mixed silage samples were assayed at Dinghu Farm of Winsor Foods Ltd. with the approval of the herbivore laboratory of South China Agricultural University (the composition and major nutrients of the test cow diets are shown in Table 1). Nylon bags (size 7 × 12 cm with 300 mesh pore size) were prepared for the determination of rumen degradation characteristics of the samples as described by Hassanat et al. (Hassanat et al. 2013). Accurately weighed (7 + 0.000 1 g) of the sample to be tested and placed in a nylon bag of known weight, 3 parallels were made for each sample at each time point to be tested, and the nylon bag was sealed with a double stranded leather band tie, 6 replicates were made for each group of samples. Each 3 samples were fixed with nylon ties on the wall of a section of plastic tube, rumen degradation test based on the principle of removing the inputs separately at one time, set 8 time points were placed in the rumen at 0, 2, 4, 8, 12, 24, 36 and 48 h, respectively, of which the sample of the time point of 0 h in a 39 °C water bath for 1 h, and the other time points of the samples taken out uniformly placed under the tap water for rinsing until the liquid was clear and odorless. The nylon bags were dried in an oven at 65 °C for 48 h and weighed. The degradation rates of rumen DM, CP and NDF were determined. The Dhanoa (1988) equation was used to determine the trophic kinetic parameters: y = a + b(1-e^− ct^), where y is the rate of protein degradation at the time of incubation, a is the rapidly degradable fraction, b is the potentially degradable fraction, c is the rate of degradation of the components, and t is the time point of incubation. Effective degradation rate: ED = a + b[c/(c + Kp)]. a, b, c as above, Kp is the outflow rate, Kp = 0.031/h (Dhanoa 2010).

**Table 1:** Composition and nutrient levels of the basal diet

Items	Content
Ingredients	
Corn, % of DM	13.31
Wheat bran, % of DM	3.67
Molasses, % of DM	0.98
Soybean meal, % of DM	3.17
Distillers dried grains with solubles, % of DM	5.61
Cottonseed meal, % of DM	2.16
Corn gluten feed, % of DM	7.31
Corn germ meal, % of DM	4.62
Premix^1^, % of DM	0.49
Corn silage, % of DM	15.70
Leymus chinensis, % of DM	42.98
Total	100.00
Nutrient levels^2^	
Net Energy for Lactating /(MJ/kg)	5.44
CP	14.28
NDF	39.17
ADF	20.12
Ca	0.60
*P*	0.40

^1^. Premix: (% of DM) Ash 99.06%, Ca 14.31%, P5.54%, Mg4.88%, K 0.06%, Na 10.59%, Cl 2.89%, S 0.36%, Co 11 mg/kg DM, Cu 576 mg/kg DM, Fe 4,869 mg/kg, I 52 mg/kg DM, Mn 1,811 mg/kg DM, Se 12 mg/kg DM, Zn 1,696 mg/kg DM, vitamin A115,210 IU/ kg DM, vitamin D 46,120 IU/kg DM, vitamin E 578 IU/kg DM

^2^. The net energy of lactation was calculated value, others were measured value

### Microbial diversity analysis

An accurately weighed 10 g of the 60d fermented mixed silage sample was mixed with sterile PBS solution at 120 rpm/min for 2 h. The samples were filtered through two layers of coarse cotton cloth, then rinsed with PBS, and the filtrate was centrifuged at 12,000 rpm /min for 15 min to collect the bacterial organisms, total DNA was extracted using the E.Z.N.A.@ kit (Omega Bio-tek, Norcross, GA, US). NanoDrop2000 was used to detect the DNA concentration and purity, and 1% agarose gel electrophoresis was used to detect the quality of the DNA, extracted DNA samples using the extracted DNA as a template to amplify the 16srrna gene of V3-V4 (primers: 314 F, 5’-AACMGGATTAGATACCCKG-3’, 805R, 5’-ACGTCATCCACCTTCC-3’). Amplification was performed using PCR. The amplification procedure was as follows: pre-denaturation at 95 °C for 3 min, 27 cycles (denaturation at 95 °C for 30 s, annealing at 55 °C for 30 s, and extension at 72 °C for 30 s), followed by a stable extension at 72 °C for 10 min, and finally storage at 4 °C (PCR instrument: ABIGeneAmp chart model 9700). The PCR reaction was performed using template DNA of 20 uL, and sterile double-distilled water was made up to 20 uL (Hassanat et al. 2013). Three replicates were performed for each sample. The PCR products from each set of samples were mixed and recovered using a 2% agarose gel for on-boarding and sequencing using Illumina’s NovaSeq PE250 platform. In order to analyze the microbial community, the relationship of overlap between the PE reads obtained by sequencing was firstly spliced, and the same quality control and filtering of the sequences was performed to distinguish the samples for subsequent analysis. The OTUs were clustered from Kingdom to Genus by UPARSE 7.1 according to the principle of sequence similarity greater than 97%. Classification was performed at 6 hierarchical levels from the Kingdom based on Silva database comparison (White et al. 2009).

### Statistical analyses

Before analysis, microbiological data were transformed by log_10_ on the basis of FM. Thereafter, all data were subjected to 2-way ANOVA with the fixed effects of CA, MA and the interaction effect of CA × MA, using the GLM procedure of SAS (version 9.0, SAS Institute Inc., Cary, NC). Tukey’s test was used for significance, where *p* < 0.05 indicates a significant difference, and *p* < 0.01 indicates a highly significant difference.

## Results

### Chemical characteristics of Chinese water chestnut residue and corn gluten meal upon mixed silage

The microbial populations and chemical composition of the two raw materials before silage were analyzed in Table 2, and the DM contents of CWCR and CGM were 19.31% and 90.79%, respectively. Due to the high water content of CWCR is difficult to direct silage, the addition of CGM can effectively reduce the silage moisture, pH of both CWCR and CGM before mixed silage is greater than 6.0, NDF, ADF and WSC content in CWCR are higher than that in CGM, but CP in CGM is much higher than that in CWCR, and the combination of the two feed stuffs can provide a rapid propagation environment for the lactic acid bacteria. *Lactobacilli* were detected in CWCR at 2.23 log_10_ cfu/g FM and yeast at 2.12 log_10_ cfu/g FM, while *E. coli* was below the detection level (< 2.00 log_10_ cfu/g FM) and molds were not detected in CWCR. *Lactobacilli*, *Enterobacteriaceae*, yeasts, and molds were not detected in all of the CGM.

**Table 2:** The chemical compositions and microbiological analysis of Chinese water chestnut residue and corn gluten meal prior to mixed silage

Items	Chinese water chestnut residue(CWCR)	corn gluten meal (CGM)	CWCR + CGM
Chemical composition			
DM, % FW	19.31	90.79	31.03
CP, %DM	10.43	41.30	15.06
pH	6.57	6.49	6.56
EE, %DM	2.91	2.41	2.84
Starch	18.21	12.70	17.39
NDF, %DM	47.37	21.30	43.45
ADF, %DM	28.68	10.80	26.00
WSC, %DM	8.80	2.41	7.84
Microorganism			
LAB, log_10_ cfu/g FW	2.23	ND	< 2.00
CB, log_10_ cfu/g FW	< 2.00	ND	< 2.00
Yeast, log_10_ cfu/g FW	2.12	ND	< 2.00
Mold, log_10_ cfu/g FW	ND	ND	ND

NDF, neutral detergent fiber; ADF, acid detergent fiber; CFU, colony forming units; CP, crude protein; DM, dry matter; FW, fresh weight; LAB, lactic acid bacteria; CB, coliform bacilli; ND, not detected; WSC, water-soluble carbohydrates

### Analysis of malic acid and citric acid, chemical composition and in situ effective degradability of Chinese water chestnut residue and corn gluten meal upon mixed silage

After 60 days of silage, the chemical composition of the mixed silage of CWCR and CGM is shown in Table 3, the addition of MA and CA showed no significant difference (*p* > 0.05) on DM and NH_3_-N in the mixed silage. In comparison to the CON group, the CP content of the three treatment groups was increased by 9%, 9.9%, and 7.3%, respectively, the CA group which showed a significant difference (*p* < 0.05). The differences were not significant in MA and CA + MA groups and there was no interaction of MA × CA interaction (*p* > 0.05). Compared with CON, the difference in NDFom content between MA and MA + CA groups was not significant (*p* > 0.05), and there was a decreasing trend with NDFom content in CA group (*p* = 0.08) and no interaction of MA × CA. ADL was not significantly (*p* > 0.05) different between MA and CA treatment groups except for MA + CA treatment group which showed a significant difference with CON formation (*p* < 0.05). There was no significant difference in the content of ADFom, Cellulose, and Hemicellulose among treatments compared with CON group (*p* > 0.05). The WSC of MA-treated group, CA-treated group, and MA + CA-treated group were all significantly reduced (*p* < 0.05) compared to CON, with MA + CA being the lowest. It can be seen that the addition of MA and CA can effectively reduce the ADL and WSC content and significantly increase the CP content in mixed storage.

**Table 3:** The chemical compositions and in situ effective degradability of Chinese water chestnut residue and corn gluten meal upon mixed silage on the condition of being added to malic acid and citric acid after 60 days

Items	Treatment ^1^				SEM	*p*-Value^3^		
	CON	MA	CA	MA + CA		MA	CA	MA×CA
**Chemical composition**								
DM, % of FW	30.52	30.61	30.85	30.91	0.13	0.28	0.13	0.92
CP, % of DM	16.06^a^	17.50^ab^	17.95^b^	17.23^ab^	0.28	0.12	0.04	0.44
NDFom, of DM	36.53	36.41	35.87	35.92	1.25	0.37	0.08	0.42
ADFom, % of DM	22.76	22.35	22.43	21.98	0.74	0.77	0.12	0.28
ADL, % of DM	3.56^b^	3.18^ab^	3.13^ab^	3.06^a^	0.08	0.09	0.048	0.31
Cellulose, % of DM	19.20	19.17	19.30	18.92	0.17	0.41	0.28	0.29
Hemicellulose, % of DM	12.77	14.06	13.59	13.89	0.34	0.12	0.09	0.25
WSC, % of DM	3.21^c^	2.87^b^	2.64^b^	2.32^a^	0.12	0.04	0.02	0.48
**In situ effective degradability**								
ISDMD,^2^% of DM	36.47^a^	38.66^b^	38.32^b^	39.76^b^	0.48	0.02	0.04	0.25
ISNDFD,^2^% of NDF	16.46	17.87	17.53	18.49	0.44	0.29	0.13	0.15
ISCPD,^2^% of CP	25.65^a^	26.76^b^	26.84^b^	27.53^b^	0.27	0.01	< 0.01	0.36

NDFom, ash-free neutral detergent fiber; ADFom, ash-free acid detergent fiber on an om basis; CP, crude protein; DM, dry matter; FW, fresh weight; WSC, water-soluble carbohydrates; SEM, standard error of the mean

^a–c^Means within a row with different superscripts differ from each other (*p* < 0.05)

^1^Control = untreated feed; MA = malic acid (10 g/kg); CA = citric acid (10 g/kg);

MA + CA = malic acid (10 g/kg) + citric acid (10 g/kg)

^2^ISDMD = in situ effective DM degradability; ISNDFD = in situ effective NDF degradability; ISCPD = in situ effective CP degradability

^3^Tukey’s test was used to detect differences between means at *p* < 0.05

The addition of MA and CA showed different effects on the effective in situ degradation rates of DM, CP and NDF in mixed silage. The results of this experiment showed that the MA-treated group, CA-treated group and MA + CA-treated group showed significant differences (*p* < 0.05) in ISDMD compared with the CON group. Among them, the effect of mixed addition of MA and CA was much higher than that of single addition. In ISNDFD, none of the three treatment groups presented significant differences compared to CON group (*p* > 0.05). In ISCPD, the ISCPD of MA, CA, and MA + CA groups were significantly increased (*p* < 0.05) compared with CON group. In addition, there was no significant difference in the two-way interaction of ISDMD, ISNDFD, and ISCPD on MA × CA.

### The Analysis of malic acid and citric acid, fermentation characteristics and microbiological analysis of Chinese water chestnut residue and corn gluten meal upon mixed silage

The rate of pH decrease during mixed storage is one of the key indicators of the success of mixed storage fermentation. As can be seen from Table 4, the pH of each treatment group with MA or CA showed a highly significant decrease compared with that of the CON group (*p* < 0.01). Among them, the MA + CA group had the lowest pH, but the difference between the three treatment groups was not significant and there was no interaction effect (*p* > 0.05). It indicated that the addition of either MA or CA could reduce the pH in mixed silage rapidly, and the MA + CA group could better maintain a lower pH state at the later stage of mixed silage, which in turn improved the fermentation quality. Compared with the CON group, the dry matter recovery (DMR) of the MA, CA and MA + CA groups did not show significant differences (*p* > 0.05), but they were all higher than that of the CON group, and also MA × CA did not show an interaction effect. NH_3_-N was lower in all treatment groups than in the CON group, but compared with the CON group the three treatment groups did not show significant differences and no MA × CA interaction (*p* > 0.05). This indicates that the addition of either CA or MA is beneficial to reduce the tendency of proteolysis in the early stage of mixed storage, reduce NH_3_-N, and improve the fermentation quality of mixed storage. In terms of lactic acid, the MA + CA group had the highest lactic acid content among all the experimental groups and was significantly higher than the other treatment groups (*p* < 0.05). The same significant difference was observed in the acetic acid content of the MA treatment group and the CA treatment group compared with the CON group (*p* < 0.05). Propionic acid content decreased in all treatment groups compared to CON group, and propionic acid was not detected in the MA + CA group. Meanwhile, butyric acid was not detected in any of the groups except the CON group.

**Table 4:** The fermentation characteristics and microbiological analysis of Chinese water chestnut residue and corn gluten meal upon mixed silage on the condition of being added to malic acid and citric acid after 60 days

Items	Treatment ^1^				SEM	*p*-Value		
	CON	MA	CA	MA + CA		MA	CA	MA×CA
DMR, % of FW	96.77	97.28	97.43	97.55	0.23	0.14	0.06	0.76
pH	4.14^b^	3.91^ab^	3.82^a^	3.72^a^	0.08	< 0.01	< 0.01	0.08
Ammonia-N, % of DM	2.83	2.49	2.54	2.24	0.12	0.35	0.48	0.92
Lactic acid, % of DM	4.72^a^	6.15^b^	6.02^b^	6.43^c^	0.55	< 0.01	< 0.01	0.42
Acetic acid, % of DM	0.73^a^	0.91^b^	0.89^b^	0.97^b^	0.09	0.03	0.01	0.57
Propionic acid, % of DM	0.11	0.08	0.05	ND	-	-	-	-
Butyric acid, % of DM	0.13	ND	ND	ND	-	-	-	-
**Microorganism**								
LAB, log_10_ cfu/g FW	5.15^a^	6.54^bc^	6.24^b^	7.89^c^	0.31	0.03	0.04	0.78
CB, log_10_ cfu/g FW	2.33	< 2.00	ND	ND	-	-	-	-
Yeast, log_10_ cfu/g FW	< 2.00	ND	< 2.00	ND	-	-	-	-
Mold, log_10_ cfu/g FW	< 2.00	ND	ND	ND	-	-	-	-

^1^Control = untreated feed; MA = malic acid (10 g/kg); CA = citric acid (10 g/kg);

MA + CA = malic acid (10 g/kg) + citric acid (10 g/kg)

^a–c^Means within a row with different superscripts differ from each other (*p* < 0.05)

As shown in Table 5, the LAB colony forming units (CFU/g) of each experimental group after mixed silage were 5.15 log_10_ cfu/g FM, 6.54 log_10_ cfu/g FM, 6.24 log_10_ cfu/g FM and 7.89 log_10_ cfu/g FM, respectively. The *E. coli* colony forming units were detected in the CON group with a value of 2.33 log_10_ cfu/g FM, *E. coli* was not detected in the CA and MA + CA groups, and *E. coli* was detected in the MA group but below the detection level (< 2.00 log_10_ cfu/g FM). Yeast was not detected in both treatment groups with the addition of MA, while it was below the level of detection in the CON and CA groups (< 2.00 log_10_ cfu/g FM). Mold production was not detected in all treatment groups except for the CON group where mold was below the level of detection (< 2.00 log_10_ cfu/g FM).

**Table 5:** Diversity of alpha index values

Sample ID	Treatment ^1^				SEM	*p*-Value		
	CON	MA	CA	MA + CA		MA	CA	MA×CA
Shannon Index ^2^	2.96^a^	3.01^a^	3.54^b^	3.78^b^	0.1	0.44	< 0.01	0.71
Chao1 Index ^2^	183.30	219.03	218.28	220.57	16.51	0.52	0.48	0.92
Simpson Index ^2^	0.82^b^	0.80^b^	0.69^a^	0.67^a^	0.07	0.03	0.34	0.61
Good’s Coverage ^2^	0.99	0.99	0.99	0.99	-	-	-	-

^1^Control = untreated feed; MA = malic acid (10 g/kg); CA = citric acid (10 g/kg);

MA + CA = malic acid (10 g/kg) + citric acid (10 g/kg)

^2^Chao was used to estimate the number of OTUs contained in the sample using the Chao algorithm; Simpson was used to estimate one of the microbial diversity indices in the sample; Shannon was used to estimate one of the microbial diversity indices in the sample

^a–c^Means within a row with different superscripts differ from each other (*p* < 0.05)

### Analysis of malic acid and citric acid in the microbial communities of Chinese water chestnut residue and corn gluten meal upon mixed silage

**Fig. 1:**
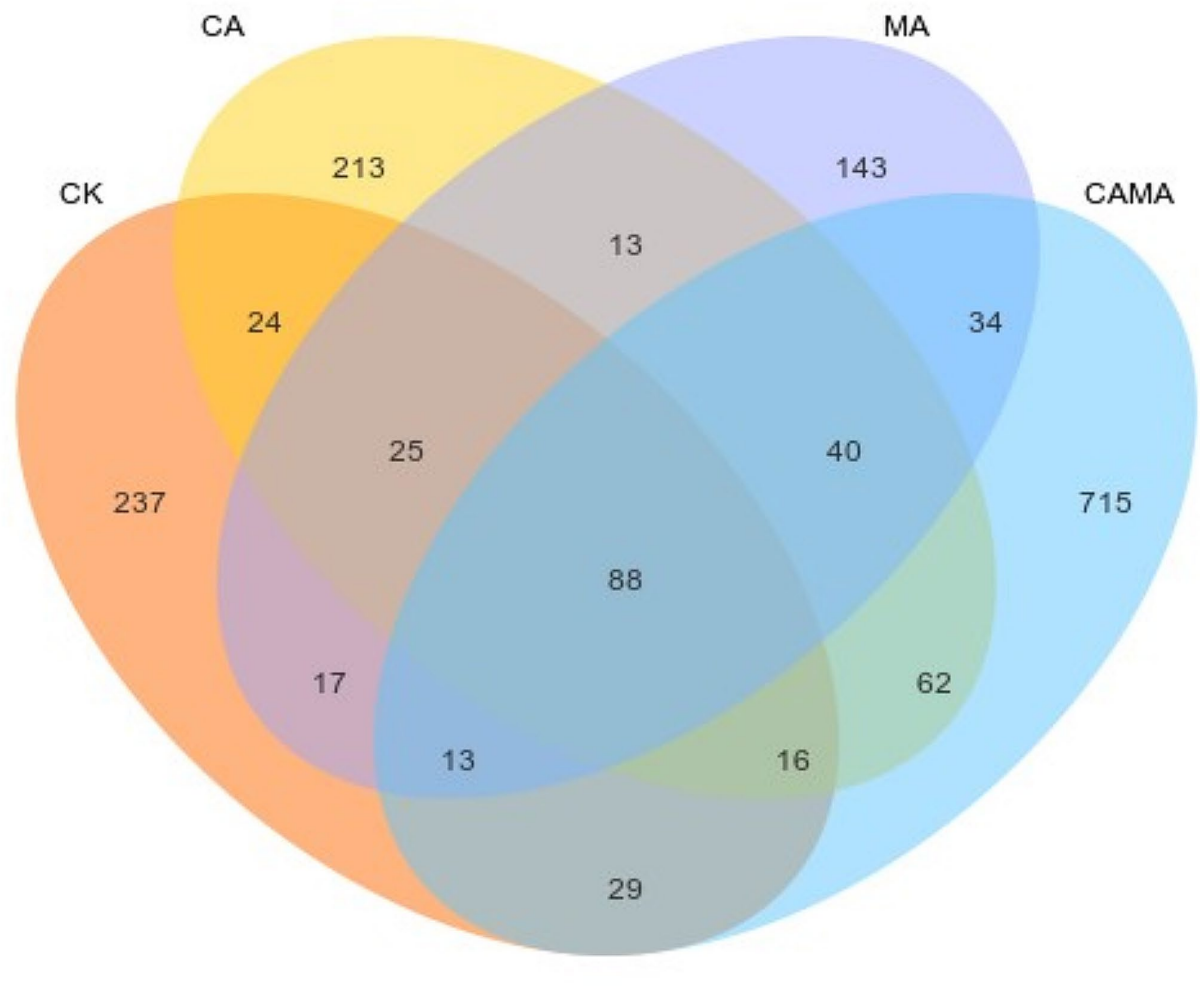
OTUs analysis of chinese water chestnut residu and corn gluten meal mixed silage bacterial community by malic acid and citric acid. (Control = untreated feed; MA = malic acid (10 g/kg); CA = citric acid (10 g/kg); MA + CA = malic acid (10 g/kg) + citric acid (10 g/kg); 1, 2, 3, three replicates for each treatment.)

**Fig. 2:**
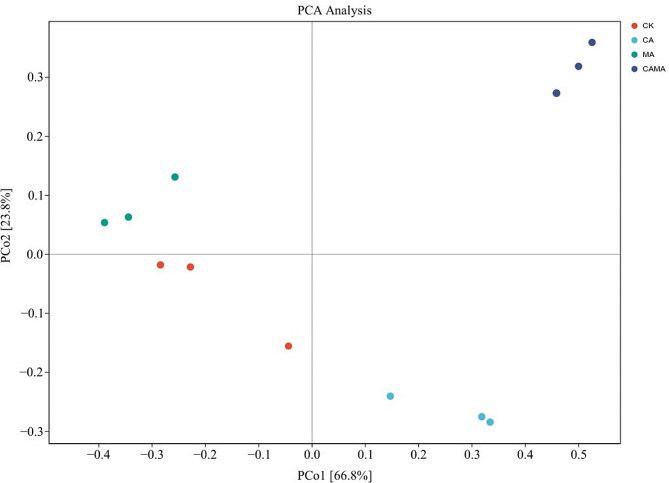
Principal component analysis of Chinese water chestnut residue and corn gluten meal mixed silage bacterial community by malic acid and citric acid. (Control = untreated feed; MA = malic acid (10 g/kg); CA = citric acid (10 g/kg); MA + CA = malic acid (10 g/kg) + citric acid (10 g/kg); 1, 2, 3, three replicates for each treatment.)

Venn diagram based on OTUs showed that a total of 14,235 OTUs were obtained from 16 S r DNA gene sequencing, and a total of 6498 OTUs were obtained among the four groups, and the OTUs specific to the CON, CA-treated, MA-treated, and MA + CA-treated groups were 237, 213, 143, and 715, respectively (Fig. 1). As shown in Table 5, there were differences in Alpha diversity among the experimental groups according to the 97% sequence similarity level after 60 d of fermentation, where the Shannon index of mixed silage in the CA and MA + CA groups increased by 19.6% and 27.7%, respectively, compared to the CON group (*p* < 0.05), and there was no significant difference between the MA group and the CON group (*p* > 0.05). Simpson’s index showed significant difference (*p* < 0.05) in MA and MA + CA groups compared to CON group, but not in CA group (*p* < 0.05). The addition of MA and CA had no significant effect on Chao index (*p* < 0.05), but it can be seen that Chao index increased after additive treatment, and the coverage of all samples was above 0.99, indicating that the results of this sequencing can reflect the real situation of the samples. Based on the weighted principal coordinate analysis of Unweighted Uni Frac (Fig. 2), the contribution rate of principal component 1 (PCo1) was 66.8%, and the contribution rate of principal component 2 (PCo2) was 23.8%. There was a difference in the microbial composition of the mixed silage in the MA group and the MA + CA group, and the mixed silage with the addition of MA could be better polymerized, the similarity of the microbial community had a differences.

**Fig. 3:**
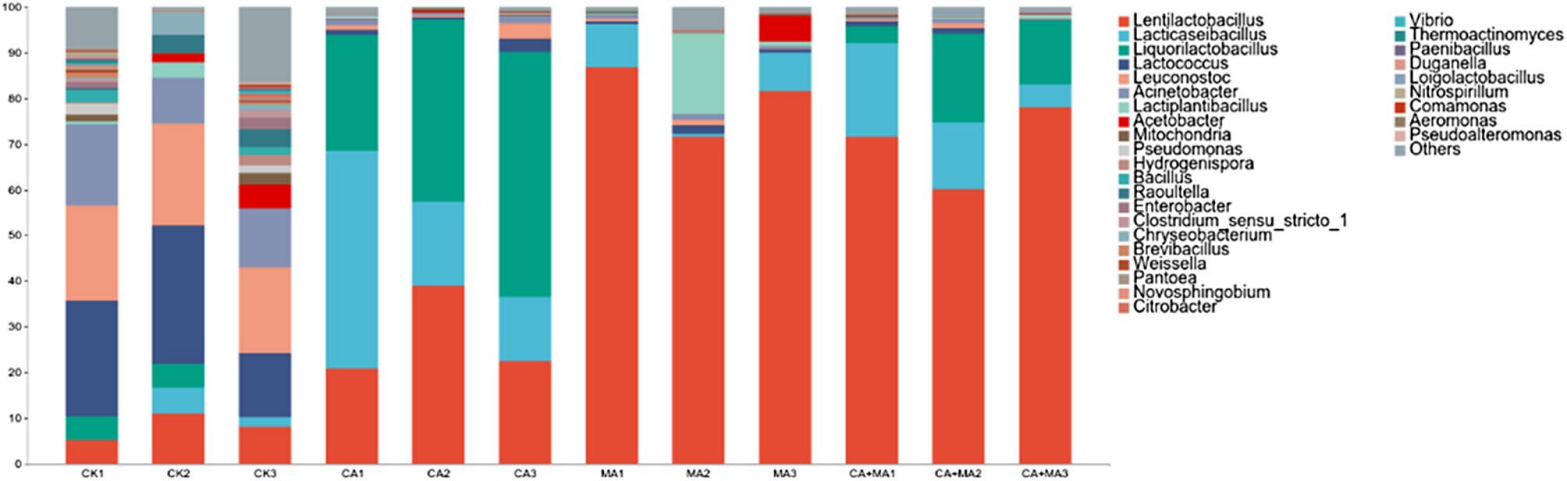
Malic acid and citric acid in the bacterial community and the relative abundance of Chinese water chestnut residue and corn gluten meal in mixed silage. (Control = untreated feed; MA = malic acid (10 g/kg); CA = citric acid (10 g/kg); MA + CA = malic acid (10 g/kg) + citric acid (10 g/kg); 1, 2, 3, three replicates for each treatment.)

To further investigate the dynamic changes of bacterial community composition at the genus level during the addition of MA and CA mixed silage, as shown in Fig. 3, bacteria at the genus level in the mixed silage samples fermented for 60 d mainly included *Lentilactobacillus*, *Lacticaseibacillus*, *Liquorilactobacillus*, *Lactococcus*, *Leuconostoc*, *Acinetobacter*, and *Lactiplantibacillus*. The dominant bacteria in all four treatment groups were mainly *Lentilactobacillus* and *Lacticaseibacillus*, where the abundance of dominant bacteria in the MA + CA treatment group was significantly higher than that in the other three treatment groups, with a ratio of 83.21%. The possible reason is that the addition of organic acids increases the number of beneficial microorganisms and reduces the proportion of heterotrophic bacteria, reducing the number of other microorganisms. Although the abundance of *Lactococcus* and *Leuconostoc* was at relatively high levels in the CON group (23.35%; 21.85%), the individual strains did not form a fermentation pattern dominated by a single strain. With the addition of CA and MA, the abundance of *Lactococcus* and *Leuconostoc* abundance decreased dramatically and in the MA + CA group only (1.41%; 1.72%) replaced by *Lentilactobacillus* dominated in the mixed silage (70.95%).

**Fig. 4:**
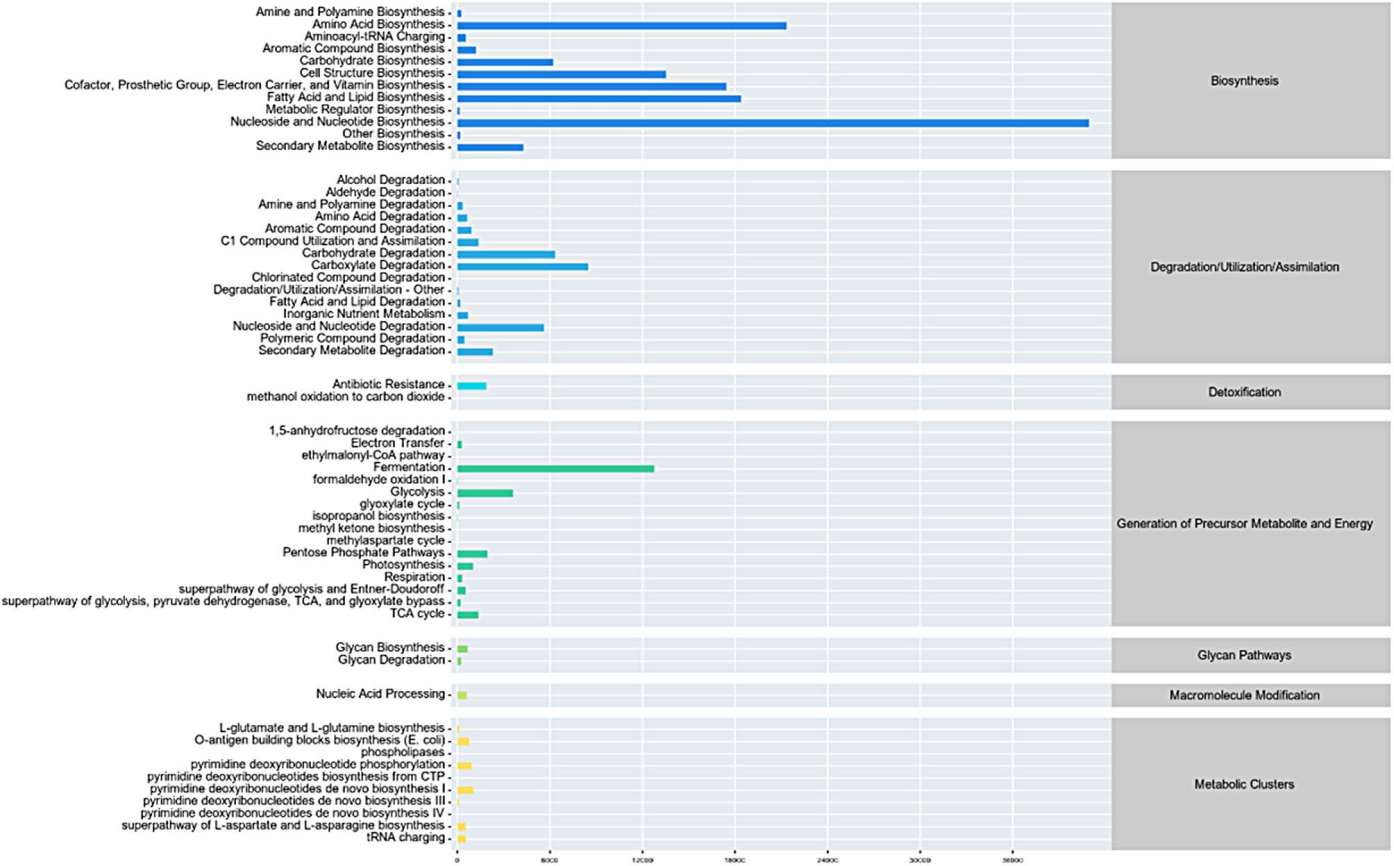
Metabolic pathway statistics analysis of Chinese water chestnut residue and corn gluten meal mixed silage bacterial community by malic acid and citric acid

**Fig. 5:**
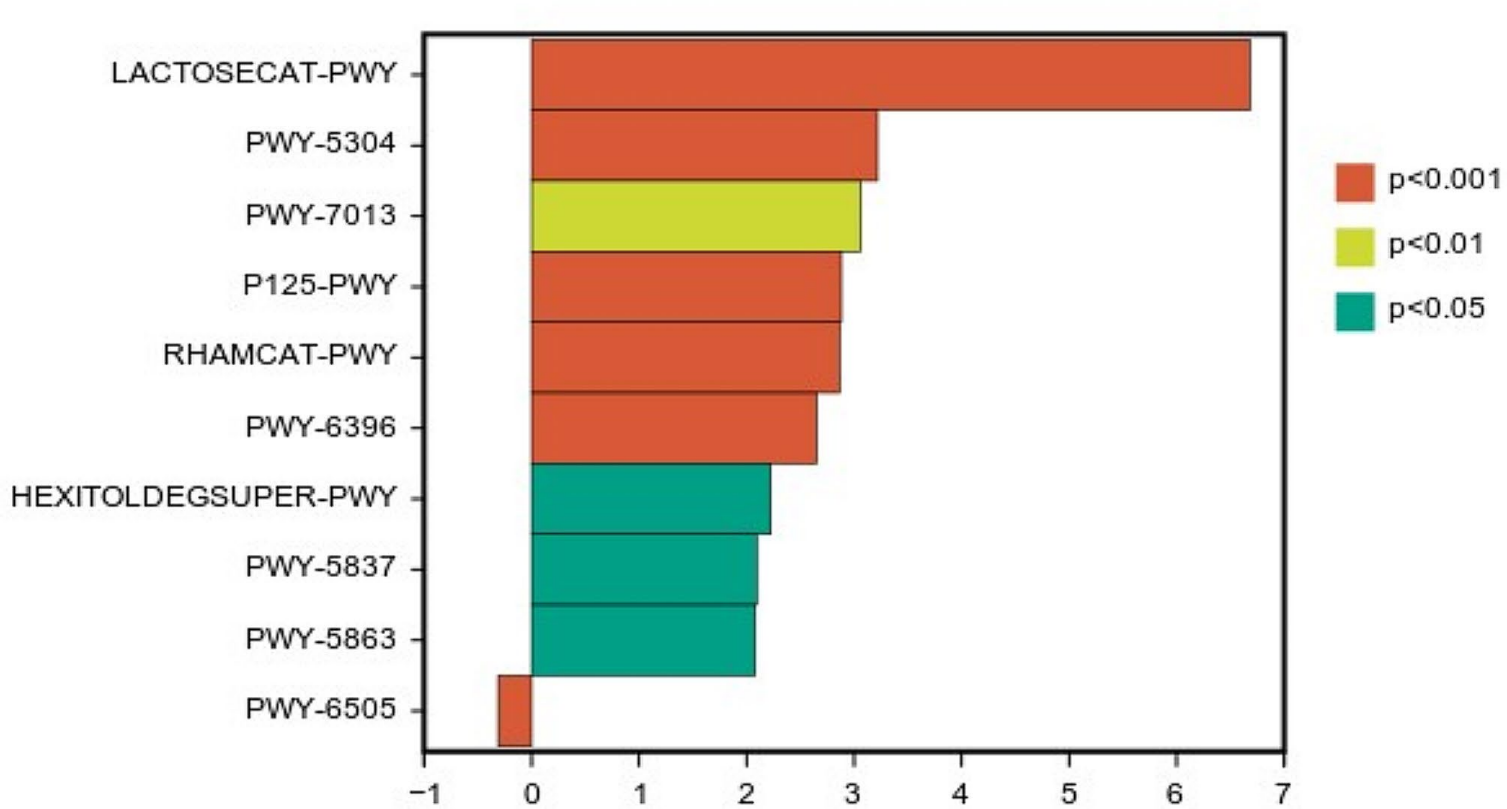
Metabolic pathway differential analysis statistics of Chinese water chestnut residue and corn gluten meal mixed silage bacterial community by malic acid and citric acid

*PICRUSt2* was utilized as an analytical tool to predict the function of CWCR and CGM mixed silage flora. The results are shown in Fig. 4. The main metabolic pathways in the first layer were Biosynthesis, Degradation/Utilization/Assimilation, Detoxification, Generation of Precursor Metabolite and Energy, Glycan Pathways, Macromolecule Modification, and Metabolic Clusters. The first 10 major metabolic pathways in the second tier are composed of: Nucleoside and Nucleotide Biosynthesis, Amino Acid Biosynthesis, Fatty Acid and Lipid Biosynthesis, Cofactor, Prosthetic Group, Electron Carrier, and Vitamin Biosynthesis, Cofactor, Prosthetic Group, Electron Carrier, and Vitamin Biosynthesis. Group, Prosthetic Group, Electron Carrier, and Vitamin Biosynthesis, Fermentation, Carboxylate Degradation, Carbohydrate Degradation, Carbohydrate Biosynthesis, Carbohydrate Biosynthesis. The nine up-regulated metabolic pathways of METACYC obtained by LEfSe analysis of the predicted pathways were LACTOSECAT-PWY, PWY-5304, PWY-7013, P125-PWY, RHAMCAT-PWY, PWY-6396, HEXITOLDEGSUPER-PWY, PWY-5837, PWY-5863. One down-regulated metabolic pathway of METACYC was obtained: PWY-6505 (Fig. 5.). The corresponding metabolic pathway numbers were taken from the METACYC database (https://metacyc.org/).

## Discussion

Usually, moisture is one of the most important factors in silage quality. The level of moisture can directly affect the propagation of microorganisms, thus influencing the silage quality (Hu et al. 2009). Meanwhile, silage raw material with less than 30% dry matter is high moisture content silage raw material (Colombari et al. 2001). An overly low dry matter content will decrease the amount of free water, reduce the rate and volume of fermentation, and raise the pH (Muck 1987). According to the results of this study, CWCR had a moisture content of 80.69%. However, when CWCR and CGM were combined, the moisture content dropped to 70%, which is consistent with previous studies on the ideal moisture level for silage (Liao et al. 2023). The results of Ni et al. showed that WSC content needs to be greater than 5% for the preparation of high quality silage (Ni et al. 2017). Although the microbiological need for WSC was higher than the WSC content of CGM in this study (2.41%), the demand of WSC for premium silage ingredients may be satisfied by mixing CWCR with CGM.

CA and MA, as an important organic acid in the tricarboxylic acid cycle, are widely used in medical, food and other fields due to their low cost. Their application as silage fermentation additives has the potential to improve silage safety and feed conversion. Meanwhile, effectively acidify silage fermentation materials can inhibit the growth of undesirable bacteria (Hu et al. 2019; Melaku et al. 2021), promoting butyric acid production leads to abnormal lactic acid fermentation, which in turn causes the mixed silage’s proteins to break down into NH_3_-N and lose silage nutrients. In this experiment, the addition of CA and MA didn’t significantly affect the CP content of mixed silage between treatment groups, probably because the NH_3_-N content was low in three treatment groups, and thus didn’t significantly affect the protein content.

pH is an important indicator for evaluating the quality of silage fermentation (Muck 2013). Its variation depends on silage dry matter, chemical composition and silage cycle. Guo et al. found that the addition of MA and CA could significantly reduce pH in alfalfa silage, and thus promote aerobic stability and fermentation quality (Guo et al. 2020). In this experiment, the pH of the treatment groups with MA and CA was below 4.00, probably because the addition of organic acids was able to rapidly reduce the pH at the beginning of silage and inhibit the growth of harmful bacteria. The linear decrease in pH also inhibits the activity of spoilage bacteria and protein hydrolysis. Lactic acid is a key indicator of silage success and one of the most important factors for good silage storage in the later stage (Liu et al. 2016). In this experiment, the content of LA increased significantly in the stage of silage fermentation up to 60 d. The likely reason for this is that the period is anaerobic fermentation stage, which favours the growth and multiplication of lactic acid bacteria (Ke et al. 2017). It has been proved that AA is also one of the important factors leading to the decrease of pH of silage, and the lower content of AA in this study may be caused by the low number of parthenogenetic lactic acid bacteria or heterogeneous lactic acid bacteria (Ertekin and Kızılşimşek 2020). At the same time, no butyric acid was found in both CA and MA treatment groups in this study, indicating that the mixed silage was of good quality and didn’t produce spoilage. Yeast and Modls in silage are closely related to silage quality. Under aerobic exposure environment, dormant aerobic microorganisms such as Yeast will utilize sugars, organic acids, amino acids and other substances produced by silage fermentation to increase silage pH, NH_3_-N content, resulting in a serious loss of DM and exacerbating silage spoilage (Chen et al. 2017). Organic acid is an aerobic microbial inhibitor, which can effectively inhibit the activity of mold and Yeast (Kleinschmit et al. 2005). The results of this experiment showed that the addition of CA and MA effectively reduced the production of undesirable microorganisms in silage. Carvalho et al. showed that the addition of 1% propionic acid significantly inhibited Yeast activity in sugarcane silage (Carvalho et al. 2012), which was consistent with the results of this study.

For the preservation of high-moisture forage, reducing the loss of forage nutrients and dry matter is crucial (Meteer et al. 2018). In this experiment, the DM of mixed silage with the addition of two organic acids, CA and MA, didn’t show significant differences, but both of them were higher than the CON group, and it can be concluded that CA and MA have the potential to reduce the DM loss of mixed silage. Lv et al. added CA to Amomum villosum silage and found that it could reduce the loss of dry matter, NH_3_-N concentration, pH, and coliform counts of silage (Lv et al. 2020). Ke et al. added MA and CA to alfalfa silage and found that both of them could effectively reduce the loss of DM and improve fermentation quality (Ke et al. 2017). And CA was more effective, which was similar to the results of this experiment. WSC is the main energy source of lactic acid bacteria during silage, and its content directly affects the production rate and content of LA, so the reduction of WSC is a normal phenomenon during silage. The WSC content was high in the early stage of fermentation, and the lactic acid bacteria multiplied rapidly in this stage, which led to the rapid decrease of pH and inhibited the growth and reproduction of undesirable microorganisms (Li et al. 2020). The WSC content in three treatment groups showed a significant decrease compared to the CON group, which is probably due to that the addition of MA and CA caused a rapid drop in pH in the early stages of silage, and at the same time promoted the multiplication of lactic acid bacteria in silage and produced large quantities of organic acids. NH_3_-N is usually regarded as an important marker of protein decomposition in silage. The ammoniacal nitrogen content was significantly reduced in the test group compared to the control group. The results showed that the addition of MA and CA either alone or in combination could significantly improve the fermentation quality of mulberry leaves. The silicates and ADL in ADF are difficult to be digested in the rumen of ruminants. NDF, as a major component of the cell wall, is commonly found in plants. The content of NDF in silage directly affects the amount of feed intake of ruminants. Bedrosian et al. showed that there was no significant difference in the NDF content of silage with the addition of organic acids as the extension of silage time (Nazar et al. 2021). However, we found that the findings of Hristov et al. were contrary to the above conclusion (Ren et al. 2020). The reason for this may be the differences in raw materials, additives. In this study, NDF, ADF and ADL didn’t show significant differences throughout the silage process.

The in situ effective degradation is an important biological validity measure of the degree of nutrient fermentation in ruminants (Nuez-Ortín and Yu, 2010). The rumen degradation rate of roughage DM can affect the intake of DM by ruminants, and it is generally believed that DMI will increase with the promote of roughage DM degradation rate. The increase of DMI will promote the nutrient intake, which will in turn improve the animals’ production performance. The higher DM degradation rate in the rumen, the easier for silage to be digested and absorbed by animals (Yu et al. 2004). Meanwhile, the dry matter intake will be higher. In this study, the DM rumen degradation rate of mixed silage with CWCR and CGM mainly included the degradation rate of CP and NDF. The results showed that the ISDMD was significantly increased in the CA group, MA group and MA + CA group compared with the CON group, which indicated that the mixed silage of the two additives could be better utilized by the rumen microorganisms. From this result, it can be concluded that the mixed silage treated with MA and CA had lower nutrient losses, thus providing more effective substrates for rumen microbial degradation (Han et al. 2015). The rumen degradation rate of forage CP is an important parameter in the composition of new protein system of dairy cows, which is mainly affected by factors such as the time duration of rumen microbial action and the ease of degradation of feed. The longer rumen fermentation time of the feed, the higher effective degradation rate (Polan et al. 1991). Mixed silage with MA and CA significantly increased ISCPD compared to CON group, indicating that mixed silage made with the two additives can be better fermented by rumen and can be utilized as a high quality feed source. In the study on alfalfa grass silage with the addition of MA and CA, Ke et al. found that they increased rumen degradation of forage nutrients based on the improvement of silage quality (Ke et al. 2017). The results of the above studies indicate that the addition of MA and CA can improve the nutritional value of CWCR and CGM mixed silage.

During the silage process, microbial communities show various changes (Ni et al. 2018). Compared with traditional sequencing methods, high-throughput sequencing can provide more accurate results, and thus can better illustrate the changes of microbiota in silage. Chao1 index is usually used to indicate the abundance of species, and the higher value proves that there are more species in the community. While Shannon index and Simpson index are more indicative of the diversity of species in bacterial communities. Shannon index is positively correlated with the diversity of community. On the contrary, Simpson index is negatively correlated with community diversity. Meanwhile, the Goods_coverage of each sample in this experiment was about 0.99, indicating that the bacteria could be detected. In this study, it was found that the addition of CA and MA effectively increased the bacterial community diversity in mixed silage compared to the CON group. This may be due to the slow decrease in pH caused by the high moisture content in the CON group, and thus didn’t immediately inhibit microbial activity, resulting in the decrease of bacterial diversity. Meanwhile, it suggests that the addition of organic acids to mixed silage can change the microbial community in silage. The results of principal component analysis showed that the crossover and aggregation between bacterial communities in each treatment group was due to differences between bacteria. We thus concluded that the addition of MA and CA changed the bacterial communities in CWCR and CGM mixed silage. Indirectly, this accounts for the differences in fermentation quality in silage (Ni et al. 2017). In this experiment, there were big differences in the dominant microbial communities of mixed silage between each treatments.

At the genus level, *Lentilactobacillus* was the main microorganism in the MA and MA + CA treatment groups of mixed silage. However, the main genera in the CON group were composed of *Lactococcus*, *Leuconostoc* and *Acinetobacter*. *Lentilactobacillus* is one of the *Lactobacillus* species, which is mostly found in malolactic fermentation system. Malolactic fermentation converts L-MA to L-lactic acid by *lactobacilli*, which reduces the pH of silage and improves the fermentation quality (du Toit et al. 2011). In this experiment, the bacterial composition of CON group was more complex, and the rate of *Liquorilactobacillus*, *Lactococcus*, and *Leuconostoc* were both more than 15%, which may be due to that the bacterial communities in the mixed silage came from different environments and had different characteristics, or the different microbial populations on the raw materials. After the addition of MA to the mixed silage, *Lentilactobacillus* immediately dominated, so that the undesirable bacteria that existed to compete for nutrients during fermentation were suppressed. At the same time, the increase of LA and AA of *Lentilactobacillus* also proved a positive correlation between the two. This indicates that the increase of LA and AA is mainly caused by the increase in the percentage of *Lentilactobacillus*. In this experiment, the genus level bacterial community in the three treatment groups evolved into 30 genera including *Lentilactobacillus*, *Lacticaseibacillus* and *Liquorilactobacillus*. Among them, the relative abundance of *Lactobacillus* was higher, which is basically in agreement with the reports of other scholars (Li and Nishino 2011). In this experiment, the percentage of lactic acid bacteria abundance increased in the MA-treated and MA + CA-treated groups after adding both MA and CA to the mixed silage, meanwhile the percentage of undesirable bacteria such as *Enterobacteriaceae*, *Salmonella* and *Campylobacter* decreased. The microbial community structure and species abundance during silage significantly affected the nutrient and fermentation quality of the forage. Prediction of bacterial community gene function in CWCR and CGM mixed silage can also indirectly reflect the fermentation quality of feeds among different treatment groups. *PICRUSt* can identify the major metabolic pathways associated with the silage process. Liu et al. utilized *PICRUSt* to study the metabolic pathways of barley silage microbial communities (Liu et al. 2019). In this experiment, the metabolic pathways of the microbial community varied due to different additives, and the relative abundance of metabolically functional microorganisms such as amino acids, carbohydrates and vitamins determined the metabolic differences of nutrients such as proteins and fibers, etc. These metabolically functional microorganisms were significantly higher in CA and MA treated groups than CON group, probably because CA and MA inhibited the decomposition of nutrients in the mixed silage. It can be concluded that the addition of CA and MA can reduce the loss of protein, carbohydrate and other nutrients in mixed silage, and this result is also consistent with the results of fermentation index and nutrient determination.

In conclusion, the results of this experiment showed that the combined addition of CA and MA could increase LA and CP content of SCWCR and CGM mixed silage and enhance the nutritional value. Meanwhile, the addition of CA and MA to SCWCR and CGM could effectively improve the rumen degradation characteristics and reduce the abundance of undesirable microorganisms. Therefore, the combination of CA and MA can significantly improve the quality of SCWCR and CGM mixed silage, making it possible to be used in dairy cattle production.

## Data Availability

The raw sequencing data from this study have been deposited in the Genome Sequence Archive in Beijing Institute of Genomics (BIG) Data Center (https://bigd.big.ac.cn/), under the accession number: CRA014288.

## References

[CR1] Broderick GA, Kang JH (1980). Automated simultaneous determination of Ammonia and total amino acids in Ruminal Fluid and in Vitro Media1. J Dairy Sci.

[CR2] Brown AT, Lee J, Adhikari R, Haydon K, Wamsley KGS (2022). Determining the optimum digestible isoleucine to lysine ratio for Ross 708 x Ross YP male broilers from 0 to 18 d of age. J Appl Poult Res.

[CR3] Cai Y, Yang J, Pang H, Kitahara M (2011). *Lactococcus* fujiensis sp. nov., a lactic acid bacterium isolated from vegetable matter. Int J Syst Evol MicroBiol.

[CR4] Carvalho BF, Ávila CLS, Pinto JC, Pereira MN, Schwan RF (2012). Effects of propionic acid and *Lactobacillus buchneri* (UFLA SIL 72) addition on fermentative and microbiological characteristics of sugar cane silage treated with and without calcium oxide. Grass Forage Sci.

[CR6] Chen MM, Liu QH, Xin GR, Zhang JG (2012) Characteristics of lactic acid bacteria isolates and their inoculating effects on the silage fermentation at high temperature. Letters in Applied Microbiology 10.1111/lam.12018 23106758

[CR5] Chen L, Yuan X-j, Li J-f, Wang S-r, Dong Z-h, Shao T (2017). Effect of lactic acid bacteria and propionic acid on conservation characteristics, aerobic stability and in vitro gas production kinetics and digestibility of whole-crop corn based total mixed ration silage. J Integr Agric.

[CR7] Colombari G, Borreani G, Crovetto GM (2001). Effect of Ensiling Alfalfa at Low and High Dry Matter on production of milk used to make grana cheese. J Dairy Sci.

[CR8] da Silva LCA, Honorato TL, Cavalcante RS, Franco TT, Rodrigues S (2012). Effect of pH and temperature on enzyme activity of Chitosanase Produced under Solid stated fermentation by Trichoderma spp. Indian J Microbiol.

[CR55] Dhanoa MS (1988) On the analysis of dacron bag data for low degradability feeds Abstract Grass and Forage Science 43(4):441–444. 10.1111/j.1365-2494.1988.tb01901.x

[CR9] Dhanoa MS (2010). On the analysis of dacron bag data for low degradability feeds. Grass Forage Sci.

[CR10] du Toit M, Engelbrecht L, Lerm E, Krieger-Weber S (2011). *Lactobacillus*: the next generation of malolactic fermentation starter cultures—an overview. Food Bioprocess Technol.

[CR11] El-Naggar A, Lee SS, Awad YM, Yang X, Ryu C, Rizwan M, Rinklebe J, Tsang DCW, Ok YS (2018). Influence of soil properties and feedstocks on biochar potential for carbon mineralization and improvement of infertile soils. Geoderma.

[CR12] Ertekin İ, Kızılşimşek M (2020). Effects of lactic acid bacteria inoculation in pre-harvesting period on fermentation and feed quality properties of alfalfa silage. Asian-Australas J Anim Sci.

[CR13] Gümü R, Ercan N, Mik H (2020). The Effect of High amounts of Wheat Gluten Meal and Corn Gluten Meal added to the diets on some serum parameters in rats. Turkish J Agric - Food Sci Technol.

[CR14] Guo X, Zhou H, Yu Z, Zhang Y (2007). Changes in the distribution of nitrogen and plant enzymatic activity during ensilage of lucerne treated with different additives. Grass Forage Sci.

[CR15] Guo XS, Bai J, Li FH, Xu DM, Zhang YX, Bu DP, Zhao LS (2020). Effects of malate, citrate, succinate and fumarate on fermentation, chemical composition, aerobic stability and digestibility of alfalfa silage. Anim Feed Sci Technol.

[CR16] Han LY, Li J, Na RS, Yu Z, Zhou H (2015). Effect of two additives on the fermentation, in vitro digestibility and aerobic security of Sorghum–sudangrass hybrid silages. Grass Forage Sci.

[CR17] Hasan MT (2015) AOAC. (1990). Official Methods of Analysis. 15th ed., Association of Official Analytical Chemists, Artington, Virginia, USA

[CR18] Hassanat F, Gervais R, Julien C, Masse DI, Lettat A, Chouinard PY, Petit HV, Benchaar C (2013). Replacing alfalfa silage with corn silage in dairy cow diets: effects on enteric methane production, ruminal fermentation, digestion, N balance, and milk production. J Dairy Sci.

[CR19] Hodge WH (1956). Chinese water chestnut or matai— A paddy crop of China. Econ Bot.

[CR21] Hu W, Schmidt RJ, McDonell EE, Klingerman CM, Kung L (2009). The effect of Lactobacillus buchneri 40788 or Lactobacillus plantarum MTD-1 on the fermentation and aerobic stability of corn silages ensiled at two dry matter contents. J Dairy Sci.

[CR20] Hu W, Li W-j, Yang H-q, Chen J-h (2019). Current strategies and future prospects for enhancing microbial production of citric acid. Appl Microbiol Biotechnol.

[CR22] Jiang H, Lu J (2018). Using an optimal CC-PLSR-RBFNN model and NIR spectroscopy for the starch content determination in corn. Spectrochim Acta Mol Biomol Spectrosc.

[CR23] Jun L, Wei H, Aili M, Juan N, Hongyan X, Jingsong H, Yunhua Z, Cuiying P (2020). Effect of lychee biochar on the remediation of heavy metal-contaminated soil using sunflower: a field experiment. Environ Res.

[CR25] Ke WC, Ding WR, Xu DM, Ding LM, Zhang P, Li FD, Guo XS (2017). Effects of addition of malic or citric acids on fermentation quality and chemical characteristics of alfalfa silage. J Dairy Sci.

[CR24] Ke W, Ding W, Xu D, Shah MN, Zhang P, Guo X (2018). Influences of addition of malic acid or citric acid, *Lactobacillus plantarum* and their mixtures on fermentation quality, proteolysis and fatty acid composition of ensiled alfalfa. Arch Anim Nutr.

[CR26] Khan TA, Nazir M, Khan EA (2013). Adsorptive removal of rhodamine B from textile wastewater using water chestnut (Trapa natans L.) peel: adsorption dynamics and kinetic studies. Toxicol Environ Chem.

[CR27] Kleinschmit DH, Schmidt RJ, Kung L (2005). The effects of various antifungal additives on the Fermentation and Aerobic Stability of Corn Silage. J Dairy Sci.

[CR30] Li Y, Nishino N (2011). Bacterial and fungal communities of wilted Italian ryegrass silage inoculated with and without *Lactobacillus rhamnosus* or *Lactobacillus buchneri*. Lett Appl Microbiol.

[CR29] Li F, Ke W, Ding Z, Bai J, Zhang Y, Xu D, Li Z, Guo X (2020). Pretreatment of Pennisetum sinese silages with ferulic acid esterase-producing lactic acid bacteria and cellulase at two dry matter contents: fermentation characteristics, carbohydrates composition and enzymatic saccharification. Bioresour Technol.

[CR28] Li F, Hu Y, Shan Y, Liu J, Ding X, Duan X, Zeng J, Jiang Y (2022). Hydrogen-rich water maintains the color quality of fresh-cut Chinese water chestnut. Postharvest Biol Technol.

[CR31] Liao Z, Chen S, Zhang L, Li S, Zhang Y, Yang X (2023). Microbial assemblages in water hyacinth silages with different initial moistures. Environ Res.

[CR33] Liu Q-h, Li X-y, Desta ST, Zhang J-g, Shao T (2016). Effects of *Lactobacillus plantarum* and fibrolytic enzyme on the fermentation quality and in vitro digestibility of total mixed rations silage including rape straw. J Integr Agric.

[CR32] Liu B, Huan H, Gu H, Xu N, Shen Q, Ding C (2019). Dynamics of a microbial community during ensiling and upon aerobic exposure in lactic acid bacteria inoculation-treated and untreated barley silages. Bioresour Technol.

[CR34] Loy DD, Lundy EL, Corn, Edition) SO, Serna-Saldivar (2019). Chapter 23 - Nutritional properties and Feeding Value of Corn and its coproducts.

[CR35] Lv H, Pian R, Xing Y, Zhou W, Yang F, Chen X, Zhang Q (2020). Effects of citric acid on fermentation characteristics and bacterial diversity of Amomum Villosum silage. Bioresour Technol.

[CR36] Melaku M, Zhong R, Han H, Wan F, Yi B, Zhang H (2021) Butyric and citric acids and their salts in Poultry Nutrition: effects on Gut Health and Intestinal Microbiota. Int J Mol Sci Vol 22. 10.3390/ijms221910392 10.3390/ijms221910392 PMC8508690 34638730

[CR37] Meteer WC, Shoup LM, Chapple WP, Meteer WT, Shike DW (2018). Effects of feeding high-moisture corn stover to gestating and lactating beef cows as an alternative to hay and corn silage on performance and reproduction. Prof Anim Sci.

[CR38] Muck RE (1987). Dry Matter Level effects on Alfalfa Silage Quality I. Nitrogen transformations. Trans Asae.

[CR39] Muck RE (2013). Recent advances in silage microbiology. AGRICULTURAL FOOD Sci.

[CR40] Nazar M, Wang S, Zhao J, Dong Z, Li J, Ali Kaka N, Shao T (2021). Effects of various epiphytic microbiota inoculation on the fermentation quality and microbial community dynamics during the ensiling of sterile Napier grass. J Appl Microbiol.

[CR41] Ni K, Wang F, Zhu B, Yang J, Zhou G, Pan Y, Tao Y, Zhong J (2017). Effects of lactic acid bacteria and molasses additives on the microbial community and fermentation quality of soybean silage. Bioresour Technol.

[CR42] Ni K, Zhao J, Zhu B, Su R, Pan Y, Ma J, Zhou G, Tao Y, Liu X, Zhong J (2018). Assessing the fermentation quality and microbial community of the mixed silage of forage soybean with crop corn or sorghum. Bioresour Technol.

[CR43] Nuez-Ortín WG (2010). Estimation of ruminal and intestinal digestion profiles, hourly effective degradation ratio and potential N to energy synchronization of co-products from bioethanol processing. J Sci Food Agric.

[CR44] Olsen RA, Bakken LR (1987). Viability of soil bacteria: optimization of plate-counting technique and comparison between total counts and plate counts within different size groups. Microb Ecol.

[CR45] Polan CE, Cummins KA, Sniffen CJ, Muscato TV, Vicini JL, Crooker BA, Clark JH, Johnson DG, Otterby DE, Guillaume B, Muller LD, Varga GA, Murray RA, Peirce-Sandner SB (1991). Responses of dairy cows to Supplemental Rumen-protected forms of methionine and Lysine. J Dairy Sci.

[CR46] Ren H, Feng Y, Liu T, Li J, Wang Z, Fu S, Zheng Y, Peng Z (2020). Effects of different simulated seasonal temperatures on the fermentation characteristics and microbial community diversities of the maize straw and cabbage waste co-ensiling system. Sci Total Environ.

[CR47] Thomas TA (1977) An Automated Procedure. For The Determination Of Soluble Carbohydrate In Herbage

[CR48] Van Soest PJ, Robertson JB, Lewis BA (1991). Methods for dietary fiber, neutral detergent fiber, and nonstarch polysaccharides in relation to animal nutrition. J Dairy Sci.

[CR49] VILA SLC, Oliveira TA, RODRIGUES AT, Tavares DC, Bongalhardo (2020). Replacement of corn and soybean meal with corn gluten meal on rooster’s diet. Turkish J Vet Anim Sci.

[CR50] White JR, Nagarajan N, Pop M (2009). Statistical methods for detecting differentially abundant features in clinical metagenomic samples. PLoS Comput Biol.

[CR51] Xu L, He W, Lu M, Yuan B, Zeng M, Tao G, Qin F, Chen J, Guan Y, He Z (2018). Enzyme-assisted ultrasonic-microwave synergistic extraction and UPLC-QTOF-MS analysis of flavonoids from Chinese water chestnut peels. Ind Crops Prod.

[CR52] You Y, Duan X, Wei X, Su X, Zhao M, Sun J, Ruenroengklin N, Jiang Y (2007). Identification of major phenolic compounds of Chinese water chestnut and their antioxidant activity. Molecules.

[CR53] Yu P, Christensen DA, McKinnon JJ (2004). In situ rumen degradation kinetics of timothy and alfalfa as affected by cultivar and stage of maturity. Can J Anim Sci.

[CR54] Zhao C, Wang L, Ma G, Jiang X, Yang J, Lv J, Zhang Y (2021) Cellulase Interacts with Lactic Acid Bacteria to Affect Fermentation Quality, Microbial Community, and Ruminal Degradability in Mixed Silage of Soybean Residue and Corn Stover. In Animals Vol. 11.10.3390/ani11020334 10.3390/ani11020334 PMC7912217 33525728

